# Somnambulists Fined

**Published:** 1860-09

**Authors:** 


*Somnambulists Fined.*—In Paris, professional somnambulists have frequently been condemned to fine and imprisonment for obtaining money on pretext of indicating occult facts. Two persons named Nicholas, man and wife, were this week committed to prison for one month, and each fined 50 fr., for swindling out of 20 fr., a man who had been robbed, and to whom they pretended falsely to indicate the thieves.

